# Perceptual asynchrony for motion

**DOI:** 10.3389/fnhum.2014.00108

**Published:** 2014-03-04

**Authors:** Yu Tung Lo, Semir Zeki

**Affiliations:** Wellcome Laboratory of Neurobiology, University College LondonLondon, UK

**Keywords:** perceptual asynchrony, motion perception, V5, visual awareness

## Abstract

Psychophysical experiments show that two different visual attributes, color and motion, processed in different areas of the visual brain, are perceived at different times relative to each other (Moutoussis and Zeki, 1997a). Here we demonstrate psychophysically that two variants of the same attribute, motion, which have the same temporal structure and are processed in the same visual areas, are also processed asynchronously. When subjects were asked to pair *up–down* motion of dots in one half of their hemifield with up-right motion in the other, they perceived the two directions of motion asynchronously, with the advantage in favor of *up-right* motion; when they were asked to pair the motion of white dots moving against a black background with that of red dots moving against an equiluminant green background, they perceived the luminant motion first, thus demonstrating a perceptual advantage of luminant over equiluminant motion. These results were not affected by motion speed or perceived motion “streaks.” We thus interpret these results to reflect the different processing times produced by luminant and equiluminant motion stimuli or by different degrees of motion direction change, thus adding to the evidence that processing time within the visual system is a major determinant of perceptual time.

## INTRODUCTION

Past work have shown that there is a perceptual asynchrony in visual perception, with different visual attributes such as color, motion and orientation being perceived at different times (Moutoussis and Zeki, 1997a, b; Barbur et al., 1998; Viviani and Aymoz, 2001; Arnold, 2005). It has been hypothesized that this asynchrony is a consequence of the fact that different attributes of the visual world are processed at different locations and take different times to completion, completion being defined as the moment when the attribute is perceived or acquires a conscious correlate (Zeki and Bartels, 1998). The method used to demonstrate perceptual asynchrony is that of pairing the properties of a single stimulus or of two stimuli. As an example, subjects may be asked to identify the color of a stimulus when it is moving upward, or the direction of motion of a moving stimulus presented in one half of the field of view with the color of a stationary stimulus presented in the other half. This is quite distinct from measuring the onset of change (temporal order judgment; Bedell et al., 2003), since in pairing experiments subjects have to determine the attributes of the stimuli over the entire presentation period, rather than the moment of change. Perceptual pairing thus measures the end result of two processing systems, and compares the time taken to perceive one attribute in relation to the time taken to perceive the other (Moutoussis and Zeki, 1997a).

If the perceptual asynchrony is the result of differences in processing time, as has been suggested (Moutoussis and Zeki, 1997b), then it should be possible to demonstrate it within a single visual domain, by manipulating stimulus parameters in such a way as to elicit different levels of excitation and inhibition from cells dealing with that domain, or by presenting stimuli to which cells respond more or less sluggishly. Indeed, Arnold and Clifford (2002) have shown that the degree of color-motion asynchrony can be manipulated and reduced by varying the angular difference in the direction of motion that is to be paired with color. Inspired by this work, we decided to go beyond and generate an asynchrony within a single visual domain, motion. When subjects were asked to pair *up–down* with *left–right* motion, the asynchrony was very close to nil (Moutoussis and Zeki, 1997b). The question that we address here, in light of the experiments by Arnold and Clifford (2002), is whether we can induce an asynchrony within the visual motion system if we vary the directions of motion to be paired. In addition, we also wanted to learn whether we could induce an asynchrony within the same system by asking subjects to pair the direction of motion of luminant dots with that of colored dots against an equiluminant colored background. The cells of area V5, specialized for visual motion (Zeki, 1974; Van Essen et al., 1981; Zeki et al., 1991), are commonly excited by motion in their preferred direction and inhibited by motion in the opposite, null, direction. The acceptance angles of such cells are wide enough so that cells that respond to motion toward, say, 12:00 o’clock will also respond when the direction of motion deviates by 15° on either side from the preferred direction, their responses beginning to fall thereafter (Zeki, 1974). Moreover, the responses of V5 cells are suppressed by prior conditioning with motion stimuli in the null direction (Priebe and Lisberger, 2002). This made it interesting to ask subjects to pair *up–down* motion presented to one visual hemifield with *up–right* motion presented to the other, with the expectation that the former will produce more inhibition than the latter, while the latter should only result in a diminution in response. Hence, we posited that when we asked subjects to pair *up–down* with *up–right* motion, the temporal perceptual advantage would be in favor of *up–right* motion. Likewise, the cells of V5 respond more sluggishly and in fewer numbers to directional motion of colored dots against an equiluminant colored background (Saito et al., 1989). Here we posited that the temporal perceptual advantage would be in favor of luminant over equiluminant motion.

We found that perception of equiluminant motion lagged behind luminant motion, and perception of opponent motion directions (*up–down*) lagged behind non-opponent motion directions (*up–right*). These results support the idea that perceptual asynchrony is a consequence of differences in processing speeds.

## METHODS

Four experiments were performed. The first was the standard one and modeled after the asynchrony experiments of Moutoussis and Zeki (1997a, b), while the other three were variations of the standard experiment (see Experiment 1), to investigate the effects of attention, motion streaks and motion speed, respectively.

### SUBJECTS

In all, 33 subjects (15 females) in the age range of 18–35 participated in these experiments, as follows: 11 participated in the standard experiment; 14 in the balanced attention version of the standard experiment; 4 in the experiment to test the effects of motion streaks on perceptual asynchrony and 4 in the test for the effect of motion speed on perceptual asynchrony. All subjects had normal or corrected-to-normal visual acuity and normal color vision as judged by the Ishihara Color Vision Test.

### MATERIALS

A 16-inch (406 mm) Sony Trinitron Multi-Scan G520 CRT screen was used for the experiment, with display dimensions 1024 × 768 pixels and refresh rate of 60 Hz. The screen was at a distance of 480 mm from the subjects, who viewed it with their heads fixed on an adjustable chin-rest and their eyes roughly level with the center of the screen. The chromaticity and luminosity of the screen were measured using a photometer (PhotoResearch Spectra-Colorimeter Model PR-670). Cogent 2000 Toolbox for MATLAB (http://www.vislab.ucl.ac.uk/cogent_2000.php) was used to execute the scripts for these experiments, which were done in a standard dark psychophysics room.

### EQUILUMINANCE DETERMINATION: HETEROCHROMATIC FLICKER PHOTOMETRY

Heterochromatic flicker photometry (Ives, 1912; Wagner and Boynton, 1972) was used. Subjects were presented with a colored rectangular field of width 45° and height 22.5°. The color of the field alternated between red and green at a frequency of 30 Hz. Subjects could adjust the RGB levels using linear sliders. The chromaticity coordinates of red, were fixed at (0.48, 0.31, 22; CIE 1931 xyY color space). Subjects were asked to adjust the green channel until they could no longer perceive the flicker. The resultant chromaticity coordinates varied slightly between subjects, from (0.26, 0.41, 21) to (0.26, 0.45, 23). At this (apparent) equiluminance point, red and green appeared to fuse into a homogenous yellowish hue. At this point, the activity of broadband phasic cells of the magnocellular pathway is reduced, while that of wavelength-selective tonic cells of the parvocellular pathway increases (Lee et al., 1988). The chromatic coordinates of the red and green values were recorded and used in the perceptual pairing experiment (see below).

### EXPERIMENT 1: PERCEPTUAL PAIRING

Here we used the same perceptual pairing method as in the original color-motion asynchrony experiment (Moutoussis and Zeki, 1997a, b) to investigate asynchrony between different types of motion stimuli. In such a design, two stimuli are displayed on the screen, either in perfect synchrony or varied in their phase (i.e., temporal synchronization) with respect to each other. A perceptual asynchrony would manifest as a consistent and reproducible perceptual bias toward one of the stimuli, resulting in mispairing of the stimuli.

**FIGURE 1:**
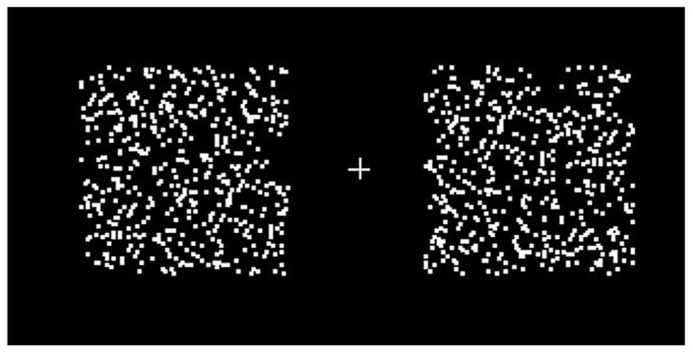
**Two moving random dot patterns were presented, one to each hemifield.** Subjects were asked to fixate the cross throughout the experiment. The random dot patterns consisted of 500 randomly scattered 0.05° squares in a 5° square region. When set in motion, the dots oscillated with 100% coherence, at a period of 833 ms. Motion directions were different for different conditions (see main text).

Two random dot patterns were presented, one in the left and one in the right visual hemifield, 3° from a central fixation cross. The patterns consisted of 500 randomly scattered 0.05° squares confined to a 5° square region (**Figure 1**). The reference motion was presented in the left hemifield, moving in an *up*–*down *square-wave oscillation; the test motion was presented in the right hemifield and moved in either an *up*-*right* oscillation (non-opponent) or a *left*–*right* oscillation (opponent; **Figure 1**). Both the reference and the test motion moved at a constant speed of 10°s^-^^1^. Subjects had to determine the direction of the test motion during the *up *phase of the reference motion.

### OPPONENT AND NON-OPPONENT MOTION

Motion in one direction is known to suppress or delay neuronal responses of a subsequent motion in the same or opposite direction (i.e., direction 180° apart) more than non-opponent motion directions (e.g., 90° apart; Snowden et al., 1991; Bradley et al., 1995; Priebe and Lisberger, 2002). The degree of inhibition increases with the angular difference and is maximal for opponent motion directions (Arnold and Clifford, 2002), with lateral inhibitory connections between direction-selective neurons being the likely basis (Snowden et al., 1991). By asking subjects to pair opponent motion (*up–down* or *left–right*) against non-opponent motion (*up-right*), we tried to create a perceptual asynchrony which is absent when subjects pair opponent motion (*up–down)* with another opponent motion (*left–right*; Moutoussis and Zeki, 1997b). Two motion settings were used, *up–down* against *left–right* (UDLR) and *up–down* against *up-right* (UDUR). In the UDLR condition, two opponent motions (*up–down *and *left–right*) were presented together. In the UDUR condition, an opponent motion (*up–down*) was presented simultaneously alongside a non-opponent motion (*up-right*). All the motion stimuli consisted of two separate motion directions alternating in a square-wave pattern. For example, *up-right* motion was achieved in two steps, first moving up then right, instead of a single diagonal movement, and similarly for the *up–down* motion and *left–right* motion.

### LUMINANT AND EQUILUMINANT CONDITIONS

Similarly, equiluminant motion reduces motion responses (Saito et al., 1989; Tootell et al., 1995). When a moving object is equiluminant with the background, and motion is solely defined by chromatic contrast, perception of motion is degraded; it is lost (Ramachandran and Gregory, 1978; Teller and Lindsey, 1993), slowed (Cavanagh et al., 1984) or fragmented (Mullen and Boulton, 1992). Brain imaging studies have found that V5 activation is significantly reduced when viewing equiluminant motion stimuli (Tootell et al., 1995). Earlier electrophysiological recordings of direction-selective neurons in macaque area V5 had also found a 35% reduction in firing rate when viewing equiluminant motion stimuli (Saito et al., 1989). In addition, retinal ganglion cells have been shown to respond more weakly to equiluminant than luminant stimuli (Kaiser et al., 1990; Valberg et al., 1992), therefore potentially conferring an early neural advantage on luminant motion. Regardless of the mechanisms or critical sites, this evidence led us to conclude that luminance-defined motion will have a processing advantage over equiluminant motion.

Three different luminance contrast conditions were used: luminant–luminant (LL), luminant–equiluminant (LE), and equiluminant–equiluminant (EE). In the equiluminant condition red squares moved against a green background of the same luminance level, determined from heterochromatic flicker photometry (Ives, 1912). In the luminant condition white squares (0.27, 0.28, 113.8; CIE 1931 xyY color space) moved against a black background (0.24, 0.26, 6.8; CIE 1931 xyY color space).

**FIGURE 2:**
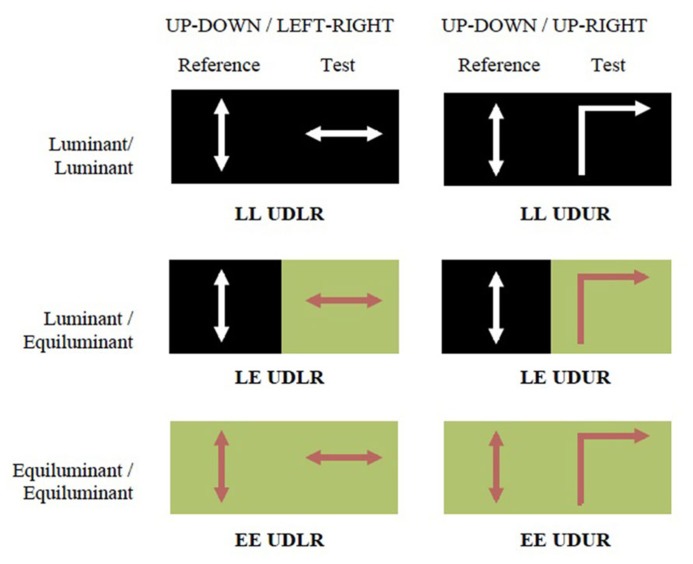
**The experiment consisted of six conditions.** The two motion settings were *up–down* against *left–right* (UDLR) or *up-right* (UDUR). The two luminance settings were equiluminant (red dots moving against equiluminant green background) and luminance (white dots moving against black background). The average luminosities of each half of the screen were roughly equal. The six conditions are abbreviated as LL UDLR, LE UDLR, EE UDLR, LL UDUR, LE UDUR, and EE UDUR.

Motion directions and luminance conditions were co-varied in six different combinations (**Figure 2**).

### OSCILLATION

**FIGURE 3:**
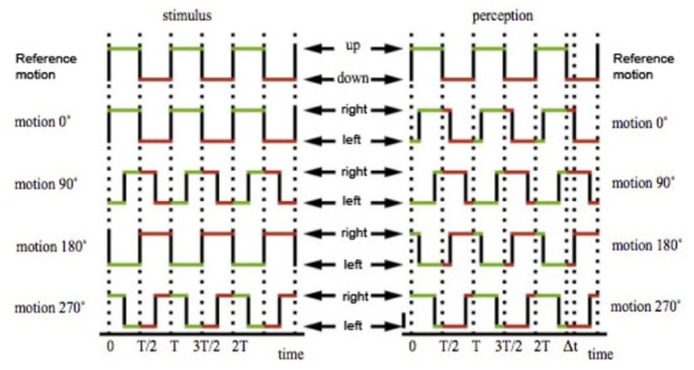
**The directions of motion of the small squares are described by two square wave oscillations, both with a period of 833 ms.** The test stimulus was shifted against the reference stimulus by a variable phase offset, ranging from 0 to 324°, in 10 steps of 36°. In temporal terms, each interval represents 83.3 ms, a tenth of a total period of 833 ms. With a 0° offset, the up phase of the reference stimulus will completely coincide with the right phase of the test stimulus. With a 180° offset, the up phase of the reference stimulus will completely coincide with the left or up phase of the test stimulus, depending on test condition. With a 90° and 270° offset, half of the up phase of the reference stimulus will coincide with half of the right phase of the test stimulus, and half with the left or up phase of the test stimulus. For simplicity, only the *up–down* against *left–right* condition is illustrated in this figure, but this also applies to the *up–down* against *up–right* condition. Figure adapted from Moutoussis and Zeki (1997a) with permission from the authors.

Motion patterns were described by square-wave oscillations of period 833 ms (i.e., 50 frames in a 60 Hz monitor; **Figure 3**). In each trial, the test stimulus was randomly shifted in time with respect to the reference stimulus by a phase difference ranging from 0 to 324°, in ten discrete steps (**Figure 3**). Each step represents an interval of 83 ms. Subjects were instructed to determine the direction of the motion of the test stimulus when the reference stimulus was on the *up* phase.

Each trial was presented for 10 s. Subjects were asked to perform a binary categorization task by identifying the predominant direction of the test motion when the reference motion was *up*. The answer could therefore be *left* or *right* in the UDLR conditions, or *up* or *right* in the UDUR conditions. For ambiguous situations, the subjects had to commit to one of the choices. The response screen remained on until subjects made a choice.

The experiment consisted of six blocks of different conditions (**Figure 2**), 160 trials per block. A blank screen was presented between trials, when subjects could take a break if they chose to. They pressed a key to proceed to the next trial. Forced breaks were enforced every 10 min, lasting for 30 s. After each block, subjects were given a 3–5 min break before continuing with the next block. They were periodically reminded to maintain fixation on the cross. The entire experiment was carried out in two separate sessions, on two different days. Each session consisted of three blocks of 160 trials. The blocks were presented in random order. At the beginning of each of the two sessions, subjects carried out 32 practice trials for the two LL conditions (LL UDLR and LL UDUR; **Figure 2**) to familiarize themselves with the experiment. The results of these practice trials were not recorded. No guidance or feedback was provided during the practice trials except to clarify the instructions.

### EXPERIMENT 2: CONTROLLING FOR ATTENTION

**FIGURE 4:**
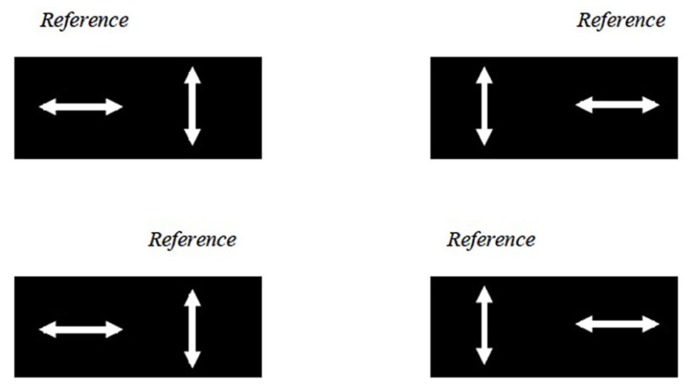
**The balanced attention version of the experiment.** Here the LL UDLR condition is used as an example. The reference motion could either be presented in the left or right half of the screen, and could either be *up–down* or *left–right* motion (and in other conditions not illustrated here, the *up-right* motion as well). These four combinations were presented in random order in four sub-blocks.

Previous studies suggest that attention speeds up the processing of a visual stimulus, thereby causing perceptual asynchrony (Clifford et al., 2003; Moutoussis, 2012). The experiment was modified to control for the effect of attention on the perceptual asynchrony observed (referred to here as the balanced attention version). The study design was identical, except that here each 160-trial block was divided into four sub-blocks of 40 trials. The reference motion could either be presented on the left or right side of the screen, and it could either be *up–down, left–right *or* up-right*. Four different combinations (**Figure 4**) were presented in random order in four sub-blocks. Before each sub-block, subjects were given a 30 s break and an instruction in the form, for example, of “UP ->LEFT/RIGHT?” on the screen. In this example, subjects had to pair the direction of the test motion (*left* or *right*) with that of the reference motion (*up*). In this setup, attention bias toward the left or right hemifield as well as toward the reference or target stimuli should balance out across the four sub-blocks.

### EXPERIMENT 3: EFFECT OF MOTION STREAKS

Amano et al. (2007) have reported that fast-moving stimuli generate “motion streaks,” leading to the appearance of orientated lines. These orientated lines in turn provide direction change cues. In this part of the experiment, in which only the luminant–luminant *up–down* against *left–right* (LL UDLR) condition was used, we wanted to investigate the effect of motion streaks on perceptual asynchrony. As a demonstration, subjects were shown a number of examples of motion streaks, with slow motion producing no visible motion streaks at a speed of 2°s^-^^1^, alongside an example of fast motion producing visible motion streaks at a speed of 60°s^-^^1^. The slow and fast motion were presented in a pair as in the main experiment (**Figure 1**), and subjects were asked to fixate the central cross throughout this part of the experiment, as before.

**FIGURE 5:**
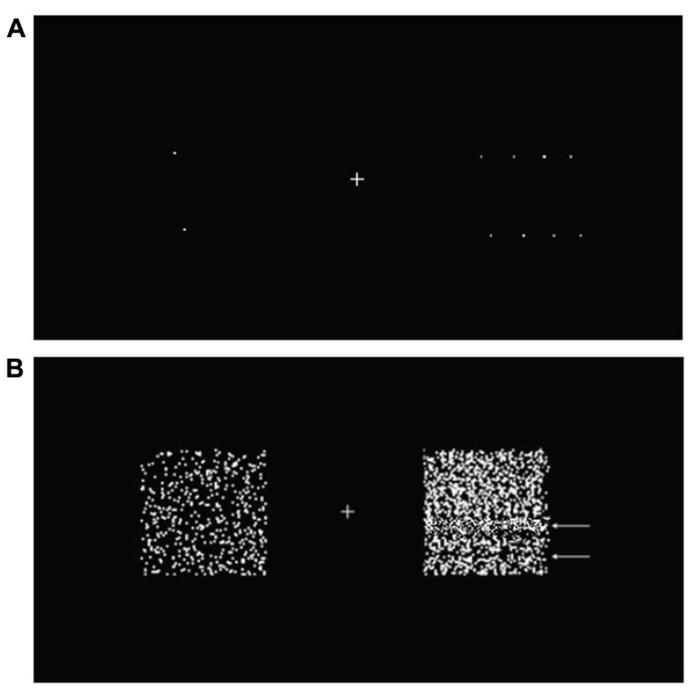
**A simulation of subjects’ perception of motion streaks.** A slow non-streaky *up–down* motion (2°/s) was presented on the left, and a fast streaky *left–right* motion (60°/s) on the right. **(A)** Two dots produced strong motion streaks. The smoothness of motion was limited by the refresh rate of the screen (60 Hz), and at very high speeds, the dots would simply “jump” from one point to another without a smooth transition. This resulted in the appearance of multiple widely spaced dots. At very high speed above 80°/s the dots appeared to merely flicker, and motion was difficult if not impossible to perceive. **(B)** For the 500-dot stimuli, banding (white arrows) could be observed in some of the fast motion trials (5°s^-^^1^ above individual subject’s threshold) due to random heterogeneity in the dot pattern. The after-images from fast motion also resulted in a denser random dot pattern as compared to the slow motion.

In order to ensure that subjects fully understood what motion streaks looked like, they were shown a slow–fast pair of stimuli consisting of an isolated dot on each side (**Figure 5A**), and were instructed to look for the appearance of a line formed by the moving dot. The initial use of an isolated dot was for the purpose of a clear, unambiguous demonstration of motion streaks, as it has been reported that motion streaks are clearer when there are fewer dots, due to the absence of significant motion deblurring (Apthorp et al., 2009). Four subsequent stimuli with more dots (2, 10, 50, and 500, with 500 being the number of dots we used in the main experiment) were then presented to acquaint subjects with what motion streaks looked like in more crowded random dot patterns, in which motion streaks were reported to be suppressed due to motion deblurring (Chen et al., 1995; Apthorp et al., 2009). Subjects were also told to look out for dark bands formed by areas of low dot density and light bands formed by areas of high dot density, as these could also provide additional spatial cues (**Figure 5B**). All other parameters were the same as the ones used in the balanced attention version of the experiment.

After the demonstration, a 1-up-1-down adaptive staircase method was used to obtain the threshold for perception of motion streaks for each subject. Subjects pressed a button when they could perceive motion streak on at least one side of the screen or if they were not certain whether a motion streak was present. The speed was then decreased by 1°s^-^^1^ in the next trial, when they pressed another button if they could not clearly see any motion streak, after which the speed was increased by 1°s^-^^1^ in the subsequent trial. 100 trials were run (except for the first subject for whom only 60 trials were run) and the speed gradually stabilized around the threshold value. The last five turning points were averaged to derive the threshold speed where motion streaks were perceptually apparent. Subjects noted that motion streaks were less obvious when dot density increased, consistent with previous reports about the greater extent of motion deblurring in dense stimuli (Chen et al., 1995; **Figure 5**).

Subjects then performed the balanced attention version of the LL UDLR and LL UDUR conditions, with two speed settings – slow and fast – for each condition. There were therefore four conditions. The speed for the slow settings was 2°s^-^^1^, whereas the speed of the fast settings was set to be 5°s^-^^1^ above the threshold value obtained in the titration for motion streak visibility. 160 trials were carried out for each of the four conditions for each subject.

### EXPERIMENT 4: EFFECT OF MOTION SPEED

While investigating motion streaks we inevitably had to vary the speed of motion, leading us to investigate whether the speed of motion has any effect on perceptual asynchrony, which was in any case an interesting problem. The LL UDLR condition was used, each subdivided into four different speed combinations: slow–slow (SS), fast–fast (FF), slow–fast (SF) and fast–slow (FS). *UD* was used as the reference stimuli for all four conditions. Slow motion was set to be 5°s^-^^1^. Fast motion was set to be 22°s^-^^1^. 40 trials were carried out for each condition.

## RESULTS

Our results show that (a) asking subjects to pair *up–down* or *left–right* motion with *up-right* directions of motion resulted in a perceptual asynchrony which was absent when they paired *up–down* with *left–right* motion, in favor of *up-right* motion. This advantage was not dependent upon the perceptual appearance of streaks and independent of the speed of motion; (b) similarly, the asynchrony was increased when equiluminant motion on one side was presented against luminant motion on the other, with the asynchrony in favor of luminant motion.

**FIGURE 6:**
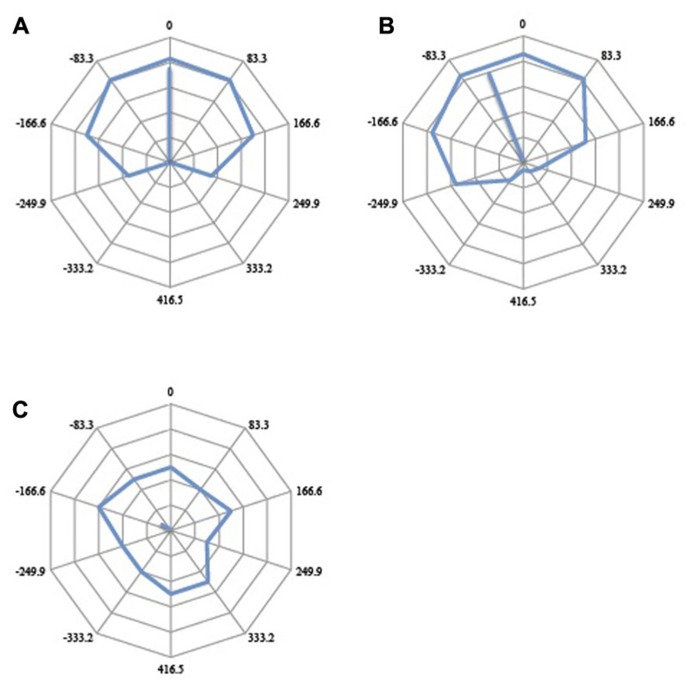
**Illustration of potential outcomes.** **(A)** The veridical response curve should the subject perceive the two motion stimuli as they happened in real time. At Oms offset, the test motion would be moving right for the entire duration that the reference motion was moving up. At 417 ms offset (half period), the test motion would be moving left for the entire duration that the reference stimulus was moving up. At 208 and -208 ms offsets (quarter period either way), the test motion would be moving right half of the time and moving left the other half of the time, or vice versa, while the reference motion was moving up. At such quarter-period offsets, if there were to be no perceptual asynchrony and subjects perceive the two motion stimuli in real time, they would judge the direction of the test motion to be either predominantly left or predominantly right, as the reference motion was moving up, with 50% probability. **(B)** If neural responses do not mirror the temporal conjunction of events as they happen in real time, the response curve would be rotated; a clockwise rotation would indicate that the test motion is perceived after the reference motion (positive perceptual asynchrony) while an anticlockwise rotation would indicate that the test stimulus is perceived first (negative perceptual asynchrony). Each data point is represented as a vector in polar space, and the average angular shift is calculated from the mean vector of 10 vectors. The angular shift quantifies the perceptual asynchrony between the reference motion and the test motion. The associated mean resultant length (MRL) of the vector quantifies the internal consistency of responses (see main text). **(C)** A short vector suggests an inconsistent and randomly distributed response. More internally consistent responses would be given more weight than inconsistent and randomly distributed responses.

**FIGURE 7:**
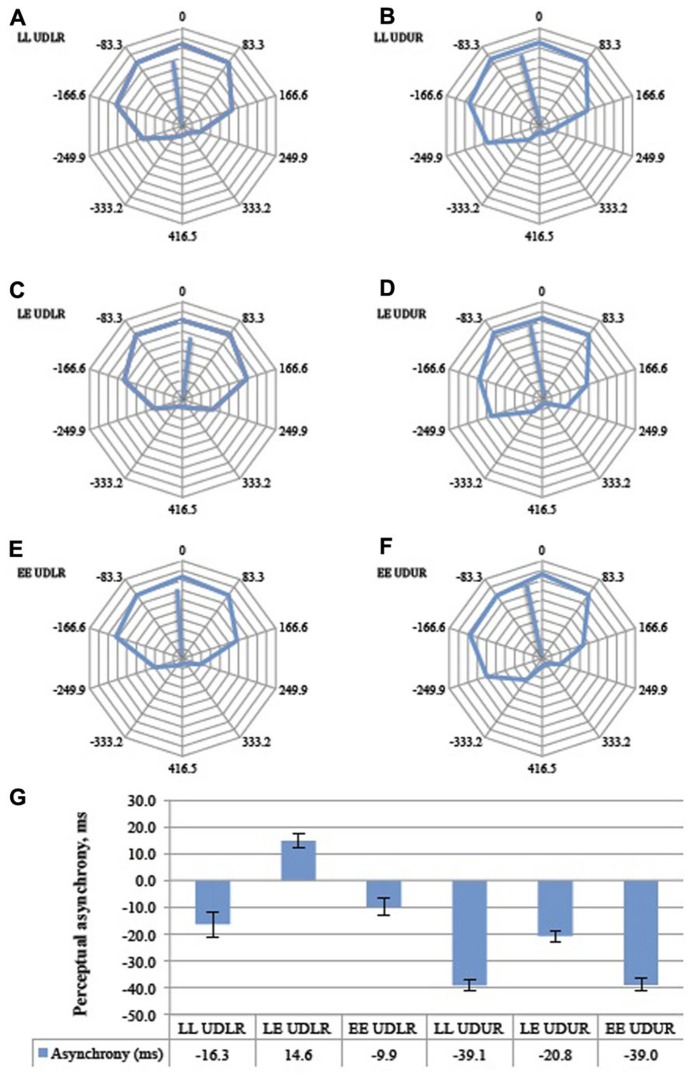
**The aggregate response curves for all 11 subjects for the six trial types (A–F).** 360° represents a period of 833 ms, and each 36° division represents 833 ms. The average angular shifts are summarized in a bar graph **(G)**. Error bars represent the standard error of the mean. LL = luminant vs. tummant, LE = luminant vs. equiluminant, EE = equiluminant vs. equiluminant. UDLR = *up–down* vs. *left–right*, UDUR = *up–down* vs. *up–right*.

The results can be represented as polar diagrams (**Figure 6**), where the frequency with which *up* is paired with *right *is plotted as a function of the phase difference between the reference and the test motion. Each data point is represented as a vector on the polar plane, and a mean resultant vector calculated by taking the mean of the 10 vectors. The angular shift of this mean resultant vector from the vertical axis represents the degree of perceptual asynchrony between the reference motion and the test motion (**Figure 6A**). Clockwise and anticlockwise rotations represent positive and negative perceptual asynchronies respectively, in milliseconds. The mean perceptual asynchrony for the 11 subjects was computed by taking the vector mean of the rotation vectors for each condition across subjects (**Figure 7**).

**FIGURE 8:**
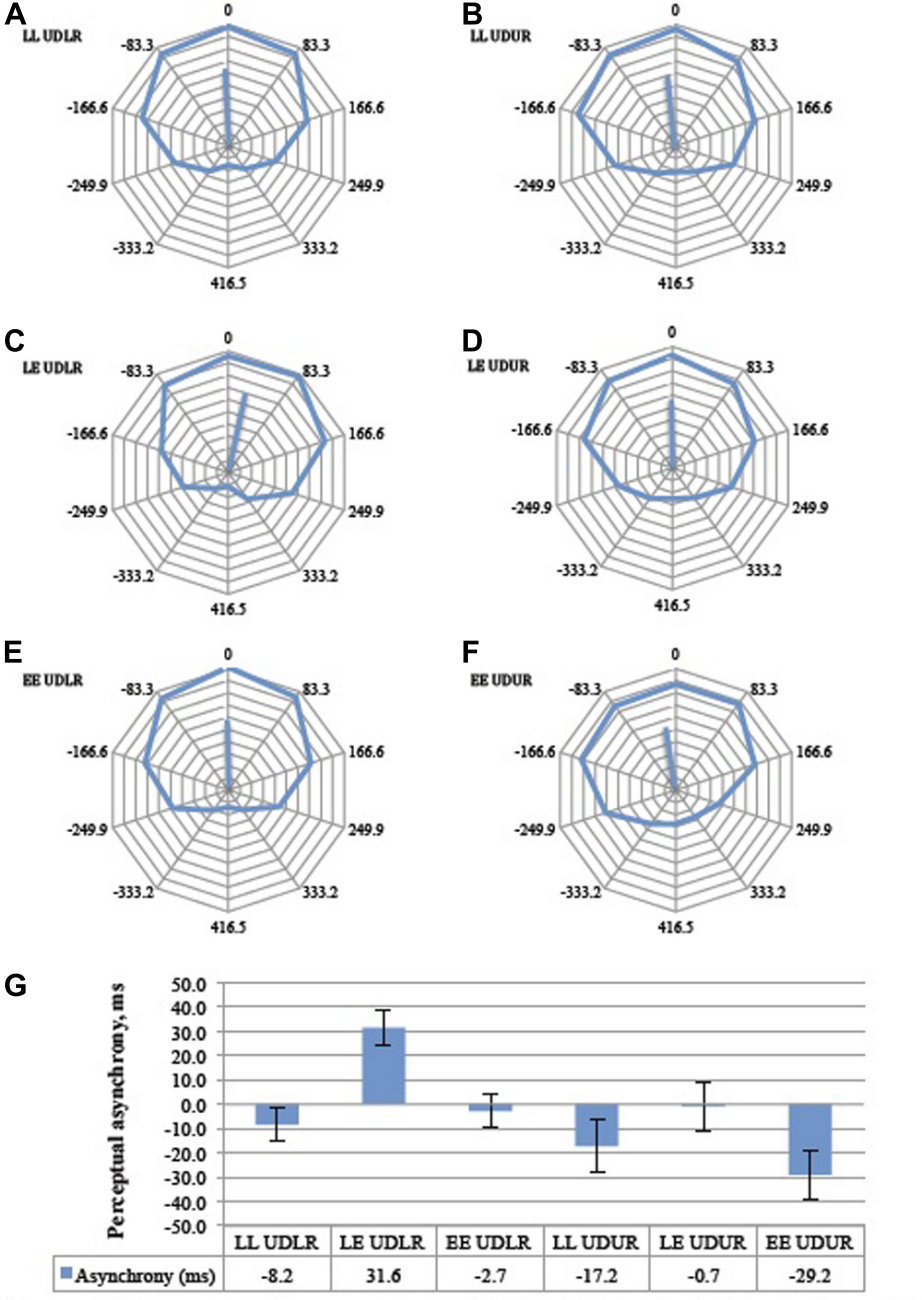
**The aggregate response curves for the balanced attention version of the experiment for all 14 subjects for the six trial types (A–F).** 360° represents a period of 833 ms and each 36° division represents 83.3 ms. The average angular shifts are summarized in a bar graph **(G)**, Error bars represent the standard error of the mean. Conventions as in **Figure 7**.

The perceptual asynchrony was assumed to follow a normal distribution and a two-way repeated measures ANOVA was conducted to test the effect of motion direction (UDLR and UDUR) and luminant/equiluminant motion (LL, LE, EE) on the degree of perceptual asynchrony. Individual responses were weighted by their corresponding vector lengths (*MRL*) to take into account the internal consistency of the responses (a response lacks internal consistency if the circular response curve is randomly distributed, with no clear preferred direction of rotation. Internal consistency is quantified by the vector length; see **Figure 5B** for an illustration). There was a statistically significant effect of motion direction [*F*(1,10) = 16.1, *p* = 0.002] and luminant/equiluminant motion [*F*(2,20) = 11.2, *p* = 0.001] on the degree of perceptual asynchrony, with no significant interaction between motion direction and luminant/equiluminant motion [*F*(2,20) = 2.03, *p* = 0.157]. In the balanced attention version of the experiment (**Figure 8**), there was a statistically significant effect of motion direction [*F*(1,13) = 12.3, *p* = 0.004] and luminance/equiluminant motion [*F*(2,26) = 13.1, *p* <0.0005] on the degree of perceptual asynchrony, with a significant interaction between them [*F*(2,26) = 3.58, *p* = 0.042].

### MOTION DIRECTION (OPPONENT VERSUS NON-OPPONENT)

*Post hoc* analysis with paired samples *t*-test adjusted for multiple comparisons using Bonferroni correction was conducted between the UDLR**(pooled LL UDLR, LE UDLR, and EE UDLR) and the UDUR (pooled LL UDUR, LE UDUR, and EE UDUR) conditions. In the first experiment, the degree of perceptual asynchrony was larger for UDUR, in favor of *up-right*, than that of UDLR**(mean difference = 29 ms, *p* = 0.002). In the balanced attention version of the experiment, the mean difference was reduced slightly to 23 ms, but was still statistically significant (*p *= 0.004). These results indicate that *up-right* was consistently perceived before *up–down*.

### LUMINANT VERSUS EQUILUMINANT MOTION

*Post hoc* analyses with paired samples *t*-tests adjusted for multiple comparisons using Bonferroni correction were conducted between the LL (pooled LL UDLR and LL UDUR), LE (pooled LE UDLR and LE UDUR), and EE (pooled EE UDLR and EE UDUR) conditions. In the first experiment, the perceptual asynchronies observed in LL conditions did not differ significantly from the EE conditions (mean difference = 4 ms, *p* > 0.500), signifying that as long as both sides have the same type of luminosity (i.e., both luminant motion, or both equiluminant motion), the degree of perceptual asynchrony will depend on the other factor (i.e., direction of motion change). It is therefore not the luminosity condition *per se* that resulted in perceptual asynchrony (which is what one might expect should the brain synchronize, say, the motion of two equiluminant stimuli less reliably than two luminant ones), but rather the conjunction of luminant motion on one side and equiluminant motion on the other. Indeed we observed a perceptual advantage of about 23 ms in favor of the luminant motion in the LE condition over and above that of the LL (mean difference = 25 ms, *p* = 0.017) and the EE (mean difference = 21 ms, *p* = 0.003) conditions. In other words, there was a perceptual asynchrony in favor of the luminant motion in the LE UDLR condition, as compared to no asynchrony in the LL UDLR and EE UDLR condition. Also, the perceptual advantage in favor of the *up-right *direction was nearly eliminated (0.7 ms) in the LE UDUR condition, as the disadvantaged equiluminant motion balanced out the advantaged *up-right* motion*. *Perceptual asynchrony was larger in the LL UDUR and the EE UDUR condition, as the *up-right* advantage was not similarly balanced out. In the balanced attention version of the experiment, the LL conditions were similarly not significantly different from the EE conditions (mean difference = 1.4 ms, *p *> 0.500). By rendering one side equiluminant and the other luminant, there was an additional perceptual advantage of about 31 ms, in favor of the luminant motion, over and above that of the LL (mean difference = 30 ms, *p *= 0.001) and LE conditions (mean difference = 31 ms, *p *= 0.007). 

In summary, the visual motion processing system was disadvantaged in the equiluminant condition compared to the luminant one and, correspondingly, the perception of *up–down* motion was disadvantaged when compared to *up-right* motion. Perception of equiluminant motion, which elicits a weaker neuronal response, therefore lags behind the perception of luminant motion by about 20–30 ms.

All subjects reported that they could clearly perceive and differentiate the directions of the equiluminant motion, even though some of them commented that equiluminant motion stimuli had a slight “shimmer” as they moved, or seemed to move more “sluggishly,” as though “moving against resistance,” or that equiluminant motion appeared to slow down upon direction reversal. Despite the lack of overt perceptual degradation reported in other studies of equiluminant motion (e.g., Ramachandran and Gregory, 1978; Teller and Lindsey, 1993), perceptual asynchrony could still be observed, suggesting that temporal asynchrony is a more fundamental, though subtler perceptual phenomenon.

### ATTENTIONAL EFFECTS

Some of the conditions in the two versions of perceptual pairing experiment (**Figures 7** and **8**) differed significantly. The LL UDLR, LE UDLR, EE UDLR, and the LE UDUR conditions did not differ significantly between the standard and the balanced attention versions (independent samples *t*-test with unequal variances, *p* = 0.38, 0.25, 0.27, and 0.08 respectively), while for the LL UDUR and EE UDUR conditions there was an additional statistically significant 14.3 ms and 13.5 ms perceptual advantage in favor of the test stimuli in the balanced attention version compared to the standard version (independent samples *t*-test with unequal variances, *p *= 0.0005, 0.042 respectively).

We attribute these differences in perceptual asynchrony to attentional bias, but such attentional bias was weak and inconsistent between conditions, and unlikely to account for the observed differences in perceptual asynchronies.

### EFFECT OF MOTION STREAKS

We found no evidence that visible motion streaks influenced the extent of perceptual asynchrony. The threshold speed for the perception of motion streaks for the four subjects ranged from 8 to 28 s^-^^1^.

**FIGURE 9:**
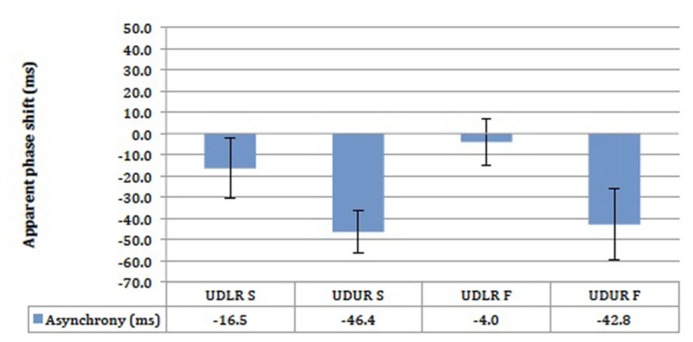
**The results of the motion-motion pairing experiment for four subjects.** UDLR = *up–down* against *left–right*; UDUR = *up–down* against *up-right*; S = slow speed setting; F = fast speed setting. Error bars represent standard error of means (SEMs).

The results did not differ significantly between the slow (2° s^-^^1^) and the fast (threshold speed +5° s^-^^1^) motion conditions (**Figure 9**). The UDLR S and UDLR F conditions did not differ significantly from 0 ms, whereas the UDUR S and UDUR F conditions did. A 30 ms difference was observed between the UDLR and UDUR conditions for both fast streaky and slow non-streaky motion. Most importantly, perceptual asynchrony could still be observed at a slow speed of 2° s^-^^1^ where visible motion streaks should be absent, suggesting that there is a perceptual advantage in favor of *up-right* motion regardless of the presence of visible motion streaks.

However, it should be noted that while we controlled for visible motion streaks to the best of our ability and ensured that they were not visible or perceptually salient, we cannot speak to the possible influence of imperceptible motion streaks on perceptual asynchrony. Due to motion deblurring, motion streaks could be rendered imperceptible even though orientation-selective neurons in the visual cortex could still be activated (Geisler et al., 2001). We nevertheless doubt that motion streaks could fully explain our results, firstly because motion streaks could not explain the perceptual asynchrony between opponent equiluminant motion and opponent luminant motion (**Figures 7** and **8**), and, secondly, it seems highly implausible that invisible motion streaks should exert a stronger effect than visible motion streaks, the latter of which we found to have no significant effect on the magnitude of the perceptual asynchrony.

### EFFECT OF MOTION SPEED

**FIGURE 10:**
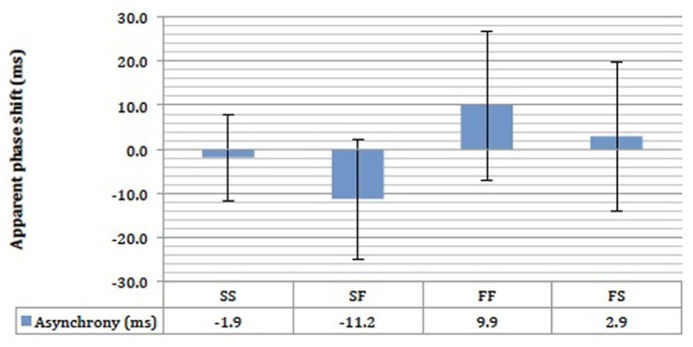
**The results of the stow motion-fast motion pairing experiment for four subjects.** SS = stow motion paired with slow motion; SF = stow motion paired with fast motion (with stow motion as the reference stimulus); FF = fast motion paired with fast motion; FS = fast motion paired with slow motion (with fast motion as the reference stimulus). Error bars represent standard error of means (SEMs).

We found no evidence that motion speed influenced the extent of perceptual asynchrony, and this is shown by the large overlap in the error bars in **Figure 10**. There was a small 11.2 ms advantage in favor of the fast motion in the SF condition, and 2.9 ms in favor of fast motion in the FS condition (**Figure 10**). Small perceptual asynchronies of 1.9 and 9.9 ms in favor of UD were observed in the SS and FF conditions respectively. None of the perceptual asynchronies was significantly different from zero. As the attention was not balanced here, these small perceptual asynchronies could be due to attentional bias. In any case, motion speed appeared not to be a significant determinant of perceptual asynchrony any more than that of endogenous attentional bias (**Figures 7** and **8**).

In summary, attentional bias played a limited role in the generation of perceptual asynchrony, and there was no evidence in our experiment that motion-derived orientation cues (arising from motion streaks) or motion speed had any effect on perceptual asynchrony. The only significant and consistently reproducible factors affecting perceptual asynchrony, in this experiment, were motion opponency (non-opponent motion perceived about 30 ms before opponent motion) and the luminance contrast of the motion (luminant motion perceived about 23 ms before equiluminant motion). We attribute the observed difference in perceptual asynchronies in our experiment to the difference in processing times, consistent with previous interpretations (Moutoussis and Zeki, 1997a, b; Arnold et al., 2001; Viviani and Aymoz, 2001; Arnold, 2005).

## DISCUSSION

In the experiments reported here, we tried to address the question of whether perceptual asynchrony can be accounted for by differences in the speed with which stimuli are processed physiologically. We chose to restrict ourselves to a single visual attribute, motion, and vary the parameters of our moving stimuli in light of previous physiological results based on the responses of cells in area V5 to opponent and non-opponent motion on the one hand, and to equiluminant and non-equiluminant stimuli on the other. Opponent motion directions strongly inhibit each other while non-opponent motion directions inhibit each other to a lesser extent (Snowden et al., 1991; Bradley et al., 1995; Priebe and Lisberger, 2002). Therefore, processing should be faster for non-opponent motion than opponent motion, leading us to perceive non-opponent motion faster than opponent motion. We indeed found that psychophysical results correspond well with these physiological findings. This result should not be affected by pairing motion stimuli moving at different speeds (within limits) because the degree of inhibition and excitation produced by stimuli of different speeds would be more or less the same, which is what we found. Similarly, equiluminant motion causes diminished response in the retina and the brain (Kaiser et al., 1990; Valberg et al., 1992), and luminant motion drives the visual motion system more strongly (Saito et al., 1989). Our results again showed that there was perceptual asynchrony in favor of luminant motion. Combining a processing advantage (non-opponent motion against opponent motion) and processing disadvantage (equiluminant motion against luminant motion) resulted in the elimination of perceptual asynchrony, as these effects canceled out (**Figure 8D**). Our perceptual pairing experiment thus demonstrated that perceptual asynchrony can be induced and its magnitude changed by changing these stimulus properties and hence the neural response to them.

The present results give rise to a testable prediction with respect to visual binding. Prior studies have shown that perceptual binding between the two different attributes of color and motion can be accelerated when the stimuli are presented with delays that compensate for the perceptual delays (Bartels and Zeki, 2006). The present results raise the question whether binding within a single attribute follows the same principle in general.

### INTERPRETATIONS OF PERCEPTUAL ASYNCHRONY

Our interpretation of perceptual asynchrony in terms of differences in processing times is not the only one. In their somewhat vague Temporal Marker Hypothesis, Nishida and Johnston (2002) have invoked the presence of hypothetical temporal markers extracted from stimuli and subsequently used to reconstruct the order of events through a separate hypothetical mechanism, leading them to argue that the color-motion asynchrony (Moutoussis and Zeki, 1997a; Viviani and Aymoz, 2001) is due to the different temporal nature of the stimuli – color being a first-order change and motion being a second-order change. What temporal marker means is not clear in neural terms, nor is there any present evidence to suggest that there exists a separate brain area or system responsible for the processing of temporal markers. However that may be, our demonstration here of a perceptual asynchrony within a single visual domain, motion, which can be induced and changed at will merely by manipulating the properties of the moving stimuli, is an argument against the Temporal Marker hypothesis. As we only used motion stimuli to demonstrate perceptual asynchrony within a single system, and because, contrary to the findings of Amano et al. (2007), we found no evidence that motion streaks and other orientation effects account for our results, we consider it unlikely that opponent motion has different temporal markers from non-opponent motion. Furthermore, it is not clear how and why luminant motion would have a different temporal marker from equiluminant motion. It is on the other hand obvious that luminant motion drives the visual motion pathway more strongly, be it at the retinal level (Kaiser et al., 1990; Valberg et al., 1992) or at higher brain areas (Saito et al., 1989), and this most likely accounted for the perceptual asynchrony between luminant motion and equiluminant motion, in favor of luminant motion. Whether the mechanism of this is due to a longer integration time in higher visual areas due to weaker signals, or an actual temporal delay in neural signal, the perception of equiluminant motion was shown here to lag behind that of luminant motion, and this disadvantage could be reduced by using the more advantageous *up-right* motion. Our demonstration adds to the views of Arnold (2005) and Moutoussis and Zeki (1997a, b) which we endorse, that the balance of evidence favors a processing time difference to account for perceptual asynchrony.

Another argument to account for the perceptual asynchrony has been attention, in particular exogenous attention. This view has been championed by Holcombe (2009) and Holcombe and Cavanagh (2008), who have demonstrated that using exogenous attention reduces or eliminates the perceptual asynchrony. Other factors may of course modulate the magnitude of perceptual asynchrony, and attention is one of them. However, as Moutoussis (2012) pointed out, an argument based on attention supports our hypothesis. Attention is widely known to modulate the responses of prestriate neurons (e.g., Chawla et al., 1999; O’Craven et al., 1999; Bichot et al., 2005), and the processing speed hypothesis predicts that if the response properties of neurons are changed, processing time will be affected, and with it the time taken to perceive the stimulus. Our main argument is simply that processing and perception of some visual attributes are independent from that of another or of others, as a result of the underlying parallel neural architecture of the visual brain and its functional specialization. We should perhaps add here that the stimuli used by Holcombe (2009) and Holcombe and Cavanagh (2008) were significantly different from ours, and it is not clear what factors influence the perception of such stimuli. It makes little sense to account for perceptual asynchrony demonstrated using one set of stimuli, processed in a given set of area(s), by using other and radically different stimuli, with other attentional demands and not necessarily processed by the same visual area(s) or systems.

It should be noted that the results obtained from perceptual asynchrony experiments cannot be compared directly to those obtained from temporal order judgment (TOJ) or reaction time experiments (see, for example, Bedell et al., 2003 and Clifford et al., 2003). The latter experiments measure perception at a specific moment in time, whereas the perceptual pairing method that we have used measures perception over a continuous span of time. It is possible and even likely that the neural mechanisms involved in perceptual pairing experiments are not the same as the ones involved in reaction time or temporal order judgments experiments. TOJ-type experiments may, for instance, involve “postdictive” mechanisms where subjects recall what they perceived after the presentation of the stimulus, whereas in perceptual pairing experiments perceptual decisions are made online while the stimulus is being shown and the subjects free to alter their judgment before committing to a decision (Moutoussis, 2012).

### THE CONSCIOUS CORRELATES OF CORTICAL ACTIVITIES

Finally, surprising though this suggestion may be, perceptual asynchrony occurring in the same visual domain shows that the activity of different (groups of) cells in the same visual domain could acquire a conscious correlate at different times. Although this remains speculative, it is consistent with the suggestion that activities in two different visual areas can acquire a conscious correlate at different times (Moutoussis and Zeki, 1997a; Zeki, 2003). But the results here highlight another issue that has been recently discussed, namely what the minimal requirement in neural size (components) for a micro-consciousness is. Escobar (2013) proposed recently that consciousness can be “quantized,” with the basic units of consciousness being products of activities in neural microcircuits. We are in general sympathetic to such a view, although what constitutes the fundamental and necessary micro-circuit remains problematic. The evidence from patients suffering from the Riddoch Syndrome (Zeki and ffytche, 1998; ffytche and Zeki, 2011), who can discriminate and are conscious of fast-moving stimuli presented to their blind fields, suggests that neither the feed-forward input from V1 to V5, nor the return input from V5 to V1 are mandatory constituents of this micro-circuit. This leaves open the question of what constituents are mandatory for such micro-circuits which allow this and other micro-consciousnesses to manifest themselves. Clearly much interesting work remains to be done to establish what the minimum requirements for a neural circuit underlying micro-coonsciousness may be. As well, the relationship between processing time and perceptual time raises important philosophical issues, including the content-vehicle distinction (Dennett and Kinsbourne, 1992). While these are fascinating issues, it is beyond the scope of this paper to consider these implications.

## Conflict of Interest Statement

The authors declare that the research was conducted in the absence of any commercial or financial relationships that could be construed as a potential conflict of interest.
